# Rheumatoid arthritis and PLA2R‐associated mebranous nephropathy. Cause or coincidence?

**DOI:** 10.1002/ccr3.9516

**Published:** 2024-11-27

**Authors:** Lara Perea‐Ortega, Ana Muñoz‐Sánchez, Myriam León‐Fradejas, Remedios Toledo Rojas, Pedro Ruiz‐Esteban, Verónica López‐Jiménez

**Affiliations:** ^1^ Nephrology Department, Regional University Hospital of Malaga University of Malaga, Biomedical Research Institute of Malaga (IBIMA)‐Plataforma BIONAND, RICORS2040 (RD21/0005/0012) Malaga Spain; ^2^ Pathological Anatomy Department ,Regional University Hospital of Malaga University of Malaga, Biomedical Research Institute of Malaga (IBIMA)‐Plataforma BIONAND, RICORS2040 (RD21/0005/0012) Malaga Spain

**Keywords:** positive antiPLA2R, primary membranous nephropathy, rheumatoid arthritis, weak apoptosis‐inducing TNF‐like (TWEAK)

## Abstract

**Key Clinical Message:**

The coexistence of rheumatoid arthritis (RA) and PLA2R‐associated membranous nephropathy (MN) is uncommon. It is difficult to demonstrate whether the mechanisms of renal pathology are triggered by RA, but it has been observed that the pro‐inflammatory molecules present in RA increase the expression of PLA2R. Rituximab could be effective in both conditions.

**Abstract:**

RA affects 0.5% of adults in our country. It is an inflammatory disease that predominantly affects the joints causing destruction of the articular cartilage. Approximately 50% of patients present extra‐articular manifestations. Renal involvement is relatively frequent and clinically significant because it worsens the course and mortality of the primary disease. The histological renal damage observed in these patients includes a wide variety of entities and histological patterns with both glomerular and tubulointerstitial involvement, with secondary MN being one of the most frequent. Coexistence with primary MN is rare. We present the case of a 46‐year‐old male recently diagnosed with RA who was referred to the nephrology department for renal function deterioration and subnephrotic proteinuria. The autoimmune study showed positive anti‐PLA2R. Due to the unusual association between both entities, it was decided to perform a renal biopsy which showed abundant spikes. The immunofluorescence study showed contiguous parietal IgG positivity (3+). Immunohistochemistry showed positive granular IgG4, confirming the diagnosis of PLA2R‐associated MN. MN is one of the most common causes of nephrotic syndrome in adults. The determination of anti‐PLA2R has been a great advance in the rapid differential diagnosis of MN. In recent years, new target antigens associated with certain underlying pathologies have been discovered. However, PLA2R is not associated with any disease or exposure and therefore remains the antigen responsible for 80% of primary NMs. Anti‐PLA2R antibodies can be produced by loss of central or peripheral tolerance. Whether these mechanisms are triggered by RA itself is difficult to prove. The cytokine TNF‐like weak inducer of apoptosis (TWEAK) has been associated with RA. This proinflammatory molecule increases the expression of PLA2R in podocytes, sensitizing them to the damaging action of anti‐PLA2Rs, which could justify a causal relationship between the two pathologies. The anti‐PLA2R positivity in a patient with membranous nephropathy should not be sufficient to refrain from searching for a secondary cause, as a kidney biopsy is mandatory when another underlying disease coexists. Treatment should be tailored to the individual risk profile for progression. Rituximab could be an optimal option for both entities.

## 1 INTRODUCTION

Rheumatoid arthritis (RA) is a chronic inflammatory disease that predominantly affects peripheral joints in a symmetrical fashion and is characterized by inflammation of the synovial membrane leading to cartilage destruction. The synovial membrane is the main focus of injury but systemic changes affecting the immune system and extra‐articular involvement can also occur.^1^ Extra‐articular manifestations occur in approximately 50% of patients, being more frequent in those with elevated rheumatoid factor (RF) titres. Renal involvement is relatively frequent and clinically significant because it worsens the course and mortality of the primary disease. It may be due to the disease itself as amyloidosis or rheumatoid vasculitis or much more frequently related to its treatment. The histological damage observed in patients with RA who have undergone renal biopsy includes a wide variety of entities and histological patterns with both glomerular and tubulointerstitial involvement. The most common are secondary membranous nephropathy (MN), membranoproliferative glomerulonephritis, minimal change disease, IgA nephropathy, analgesic nephropathy and interstitial nephritis.^2^, ^3^

Coexistence with primary MN is rare. We present a case where both pathologies are diagnosed.

## 2 CASE HISTORY AND EXAMINATION

A 46‐year‐old white male, his medical history included hypertension since the age of 40, with regular control, dyslipidaemia and arthralgias for a couple of years, for which he had been under recent follow‐up by Rheumatology, without the presence of arthritis or fever, or uveitis, or other systemic autoimmune symptoms. He was referred to the nephrology department for routine laboratory tests showing alterations in renal function with creatinine (Cr) 1.7 mg/dL and subnephrotic proteinuria with a protein/Cr ratio (CPC) 2.3 g/g, normal sediment. On reviewing previous analyses, the patient had already been in renal failure for 2 years, with Cr 1.3 mg/dL and CPC 1.7 g/g. Physical examination revealed grade II obesity with a BMI of 35 and arterial hypertension (150/90 mmHg).

The requested blood test showed a slight improvement in Cr of 1.4 mg/dL, albumin of 3.5 g/dL, lipid profile with LDL of 85 mg/dL and triglycerides of 272 mg/dL. Serology for human immunodeficiency virus and hepatic viruses were negative. Hepatic profile was normal. The immunological study showed positive anti M‐type phospholipase A2 receptor antibody (anti‐PLA2R) with a low titre in 1/10, the rest normal. Urine proteinuria of 2.7 g/24 h with normal sediment was detected. Ultrasonography showed kidneys of normal size and echostructure, without alterations in the renal Doppler. Antiproteinuric treatment was intensified with ARA2 and ISGLT2 and complementary studies were performed to rule out an underlying neoplastic process.

From the rheumatological point of view, the patient was diagnosed with RA with positive RF at 35 IU/mL and high titre anti‐cyclic citrullinated peptide antibody (anti‐CCP) at 432 U/mL. It was decided to start treatment with DMARD (methotrexate).

## 3 METHODS

**FIGURE 1:**
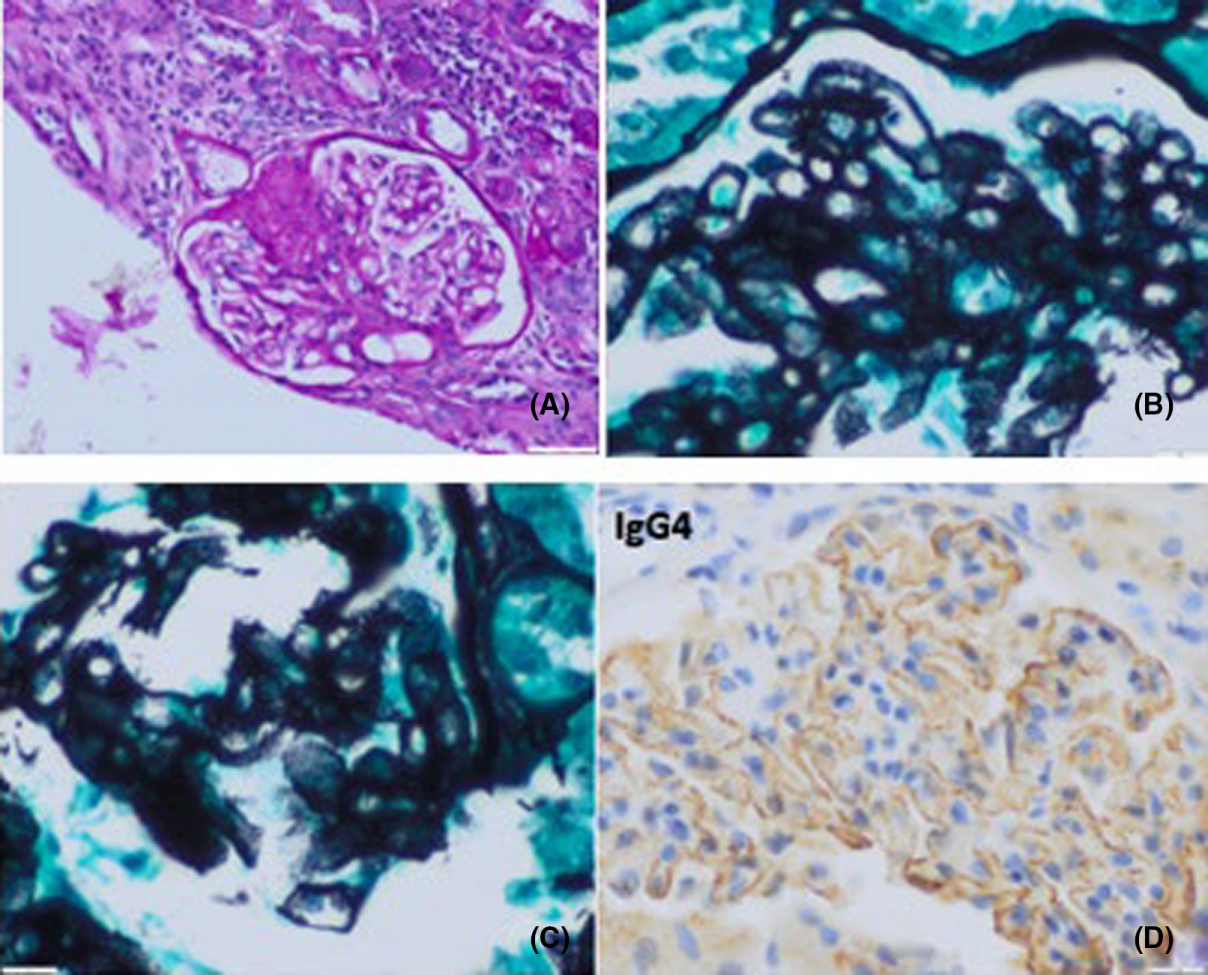
(A) Optical microscopy. HE 20x. Thickening and stiffening of the capillary walls of the ball. (B)Silver‐methanamine. Moth‐eaten appearance as vacuoles or bubbles in the capillary wall. (C) Silver‐methanamine. Spikes that prolong the glomerular basement membrane. (D)Immunohistochemistry. IgG4 positive, characteristic of primary membranous nephropathy.

Given the subnephrotic proteinuria, impaired renal function, low anti‐PLA2R titre and recent diagnosis of RA, a renal biopsy was performed. The cylinder contained 13 glomeruli, 4 of which were globally sclerosed and the rest showed thickening and rigidity of the capillary walls under light microscopy, with abundant spikes observed with the silver‐methanamine technique. Interstitium with mild infiltrate and minimal foci of interstitial fibrosis and tubular atrophy. The immunofluorescence study showed contiguous parietal positivity for IgG (3+), IgM (+), Kappa (2+), Lambda (2+), being negative for IgA, C3 and fibrinogen. Immunohistochemistry showed positive IgG4 positive parietal granular IgG4 contiguous to the glomerular level, without the option of performing the anti‐PLA2R technique in tissue. A histological diagnosis of stage II NM of primary origin was made (Figure 1).

## 4 OUTCOME AND FOLLOW‐UP

On review in consultation (3 months), an increase in proteinuria of 7.2 g/24 h, Cr by 1.7 mg/dL and a higher titre of anti‐PLA2R at 1/100, LDL 85 mg/dL, albumin 3.5 g/dL were observed. In view of this worsening, it was decided in conjunction with Rheumatology to suspend methotrexate (he was on treatment for a total of 3 months) and add Rituximab (RTX) with a schedule of two 1 g doses, separated by 15 days.

After 8 months of treatment, the patient presented partial renal remission. With proteinuria of 2 g/24 h and Cr 1.35 mg/dL, achieving negative anti‐PLA2R (Table 1). From the rheumatological point of view, he presented clinical improvement with partial serological decrease (RF at 18 IU/mL and anti‐CCP 200 U/mL). The patient is to be monitored over the next few months.

**TABLE 1:** Biochemical parameters since the start of treatment with Rituximab.

Biochemical parameter	Basal	8 months
Creatinine	1.7 mg/dL (0.7–1.3)	1.35 mg/dL
FG CKD‐EPI	44 mL/min	62 mL/min
Proteinuria /24 h	7.2 g/24 h	2 g/24 h
Sediment	Negative	Negative
Albumin	3.5 g/dL	3.8 g/dL
Anti‐PLA2R	1/100	Negative
FR	35 IU/mL	18 IU/mL
Anti‐CCP	432 U/mL	200 U/mL
VSG	24	16

## 5 DISCUSSION

MN is one of the most common causes of nephrotic syndrome in adults. The pathology of MN is characterized by a global and diffuse distribution of subepithelial deposits,^4^ leading to thickening of the capillary wall when examined with an optical microscope. The primary (idiopathic) form is associated with autoantibodies against various kidney‐specific podocyte antigens (70–80%), while the secondary form is linked to systemic diseases with renal involvement (20–30%).^5^ In recent years, target antigens associated with certain underlying pathologies have been discovered.^6^

Anti‐PLA2R antibodies have represented a significant advance in the rapid differential diagnosis of primary MN. Their sensitivity is 70%–80% and their specificity is 98%–100%. However, low titers have been detected in patients with active hepatitis B/C and sarcoidosis.^7^, ^8^ The antibodies formed against this protein, primarily IgG4, cross the glomerular capillary and bind to the protein along the external or subepithelial aspect of the capillary wall, forming the typical subepithelial deposits. A soluble form of PLA2R has been detected in the serum of mice and humans, resulting from the proteolytic shedding of membrane‐bound PLA2R.^9^ Anti‐PLA2R antibodies may arise from loss of central or peripheral tolerance, altered expression of PLA2R in podocytes, sensitizing them to the harmful action of anti‐PLA2R, or molecular mimicry.

RA is a chronic autoimmune disease characterized by inflammation and degradation of peripheral joints, affecting approximately 0.5%–1% of adults, with an incidence of 0.5% in our country. It can occur at any age, but is more common between the fourth and sixth decades of life, predominantly in females (3:1). Its etiology remains unknown, although there may be a genetic predisposition with a polygenic inheritance model (higher incidence among first‐degree relatives, monozygotic twins, and strong association with certain HLA), along with the influence of environmental factors acting as disease triggers.^3^ Furthermore, the inflammation associated with RA can damage other parts of the body. Extra‐articular manifestations occur in approximately 50% of patients, with the kidney being one of the affected organs. The cytokine network in this disease is complex, with the tumor necrosis factor (TNF) superfamily playing an important role, particularly the TNF‐like weak inducer of apoptosis (TWEAK) cytokine, which is found in high concentrations in the synovial tissue of patients with active RA.^10^

TWEAK is expressed as a membrane protein and as a soluble protein of 168 amino acids (18 kDa) that results from the proteolysis of membrane TWEAK.^11^, ^12^, ^13^ TWEAK exerts a variety of biological effects through binding to its receptor, Fn14, playing a key role as a mediator of inflammation and bone erosion in RA. Recently, serum TWEAK has been proposed as a biomarker for early diagnosis of RA.^14^

It is difficult to verify whether the MN in our patient was triggered by the RA itself. In general, anti‐PLA2R positivity is accepted as diagnostic of primary MN, given its high sensitivity and specificity, even when other conditions theoretically responsible for the process coexist in the patient.

The function of PLA2R in podocyte biology is not clear, although it may be involved in the binding or internalization of collagen through the fibronectin II domain. Genetic and environmental factors have been proposed for the formation of anti‐PLA2R. The immune system plays a key role in stimulating the production of antibodies and regulating the expression of PLA2R. Pro‐inflammatory molecules such as TWEAK have been shown to increase PLA2R expression. To understand the mechanism of action of TWEAK on the kidney, various studies have been conducted by Alberto Ortiz's research group. They found that TWEAK, through the activation of the Fn14 receptor, upregulates three non‐HLA genes (PLA2R, NFKB1, IRF4) associated with MN in podocytes, thereby increasing PLA2R expression and favoring the harmful action of anti‐PLA2R.^15^ They conclude that TWEAK could become a therapeutic target.^9^, ^15^ Recently, in another study using the STARMEN cohort, it was observed that urinary TWEAK levels could correlate with serum anti‐PLA2R titers.^16^ In line with Ortiz's findings, another French study confirmed that treating differentiated podocytes with TWEAK led to a significant increase in PLA2R expression.^17^

This could justify a causal relationship between these two entities, suggesting that the immunological environment created by RA may trigger the immune response leading to the generation of anti‐PLA2Rs and the development of primary MN. Moreover, in our patient, the parallel evolution of both the onset and clinical course of RA and MN is highly suggestive of this causal relationship.

It is difficult to predict the frequency of association between RA and PLA2R‐associated MN. To the best of our knowledge, there are few published cases where both pathologies coexist. A case of a Hispanic male with RA who developed PLA2R‐associated MN complicated by anti‐glomerular basement membrane (anti‐MBG) disease has been published.^18^ Several studies have been conducted to evaluate the causes and effects of RA on MN, but they do not include the determination of PLA2R.^19^ Many of the published case series studying the possible presence of anti‐PLA2R in patients with autoimmune diseases do not include RA. The Mayo Clinic published a retrospective study of 270 patients aimed at describing the clinical and pathological phenotype of MN associated with different antigens, including PLA2R. A total of 220 patients presented PLA2R. An associated disease was identified in 27% of the patients, of which 13% (36 patients) corresponded to autoimmune disease. Of these, only 3 patients had RA, and in one of them, PLA2R was present; however, this patient also had coexisting small cell lung carcinoma. Regarding the PLA2R antigen, they concluded that patients with PLA2R‐associated MN were predominantly middle‐aged white males without associated disease.^20^

Treatment should be adapted to the individual risk profile for progression.^21^ B lymphocytes play a nominal role in the pathophysiology of both diseases, being responsible for the production and maturation of autoantibodies such as RF and anti‐CCP or anti‐PLA2R. RTX is a chimeric monoclonal antibody that reduces the population of CD20+ B lymphocytes and is a therapeutic option for both conditions.^21^, ^22^ In our case, we decided to initiate treatment with this drug due to the moderate risk of progression of MN, and we are monitoring the evolution of both pathologies to see if it is necessary to combine it with another immunosuppressant.

In conclusion, anti‐PLA2R positivity in a patient with MN should not be sufficient to refrain from searching for a secondary cause, as a kidney biopsy is mandatory when another underlying disease coexists. New studies could shed light on the triggering mechanisms of these rare and infrequently coinciding pathologies, as well as potential therapeutic targets for RA and PLA2R‐associated MN.

## AUTHOR CONTRIBUTIONS

**Verónica López‐Jiménez:** Funding acquisition; supervision; writing – review and editing. **Ana Muñoz‐Sánchez:** Conceptualization; writing – original draft; writing – review and editing. **Pedro Ruiz‐Esteban:** Writing – review and editing. **Remedios Toledo Rojas:** Methodology; writing – review and editing. **Myriam León‐Fradejas:** Methodology; writing – review and editing. **Lara Perea‐Ortega:** Conceptualization; writing – original draft; writing – review and editing.

## FUNDING INFORMATION

This study was partly funded by grants from the Instituto de Salud Carlos III, Madrid, Spain (ICI21/00042, and RICORS Network RD21/0005/0012) and FONDOS FEDER. These institutions had no part in the design of the study and collection, analysis, and interpretation of data or in writing the manuscript.

## CONFLICT OF INTEREST STATEMENT

The authors declare that they have no competing interest.

## ETHICS STATEMENT

Medical record review was approved by the hospital ethics committee (CEI Provincial de Málaga), which followed normal clinical practice and according to the Declaration of Helsinki, and has given its written consent. The patient also gave written informed consent for all the procedures and publication of the case.

## CONSENT

The patient gave written informed consent for the publication of the case report and accompanying images. A copy of the informed consent is available for review by the editor.

## Data Availability

Data sharing is not applicable to this article as no datasets were generated or analyzed during the current study.
